# An Unusual Long-Term Survey of a Patient with Widespread Malignant Urachal Tumor, Not Given Chemotherapy or Radiotherapy

**DOI:** 10.1155/2015/183787

**Published:** 2015-03-08

**Authors:** Tugrul Ormeci, Murat Can Kiremit, Bulent Erkurt, Aslı Örmeci

**Affiliations:** ^1^Department of Radiology, Faculty of Medicine, Medipol University, Istanbul, Turkey; ^2^Department of Urology, Faculty of Medicine, Medipol University, Istanbul, Turkey; ^3^Department of Gastroenterology, Haseki Research and Educational Hospital, Istanbul, Turkey

## Abstract

The urachus establishes a connection between the dome of the bladder and the umbilicus throughout fetal life. If the urachus does not close completely, malignancy is a potential complication. The primary treatment for malignant urachal tumor is surgical excision. A 61-year-old male patient diagnosed with urachal carcinoma had undergone partial cystectomy 25 years previously. Twenty years later, local recurrence was treated with another partial cystectomy without umbilical remnant excision. Recurrence at the umbilical site was excised 2 years later, but intraperitoneal invasion had occurred, and the patient underwent a total colectomy at that time. Local disease and disseminated metastases in the thorax and intra- and extraperitoneal areas were noted upon admission to our hospital. Urachal carcinomas are usually aggressive tumors, and surgical treatment should include partial or radical cystectomy and excision of the urachus and umbilicus, to prevent local recurrence and distant metastasis.

## 1. Introduction

The urachus is the vestigial remnant of the cloaca and the allantois, forming a fibrous embryonic remnant that extends from the dome of the bladder to the umbilicus. Urachal remnants may be noted in infants and during childhood, where they are most frequently found as thin-walled, internally homogenous cysts, containing straw yellow umbilical fluid. The development of malignancy is noted in adults. Urachal carcinomas constitute about 0.2% of all bladder tumors [1]. The primary treatment for malignant urachal pathologies is surgical excision.

Herein we reported an unusual case of a patient with an inadequately treated urachal carcinoma who did not receive chemotherapy or radiotherapy, over a period of 25 years.

## 2. Case Presentation

**Figure 1:**
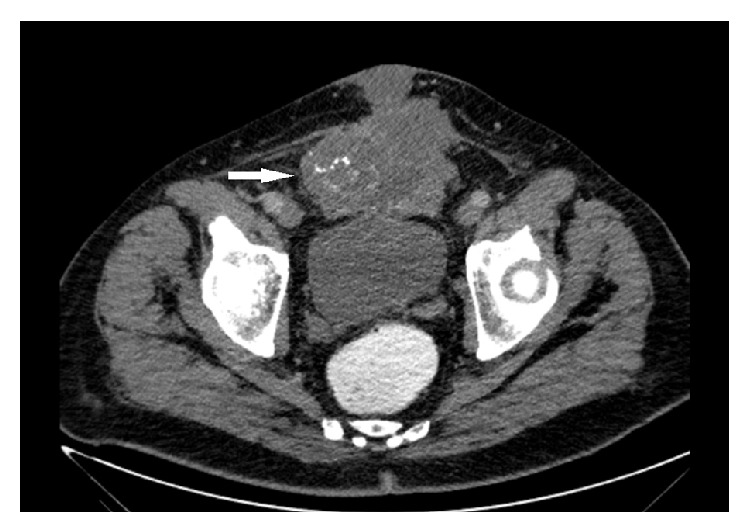
Cross-section contrast-enhanced computed tomography (CECT). There is a heterogeneous lesion (arrow) filling the bladder, extending to the anterior, and containing punctate amorphous calcifications.

**Figure 2:**
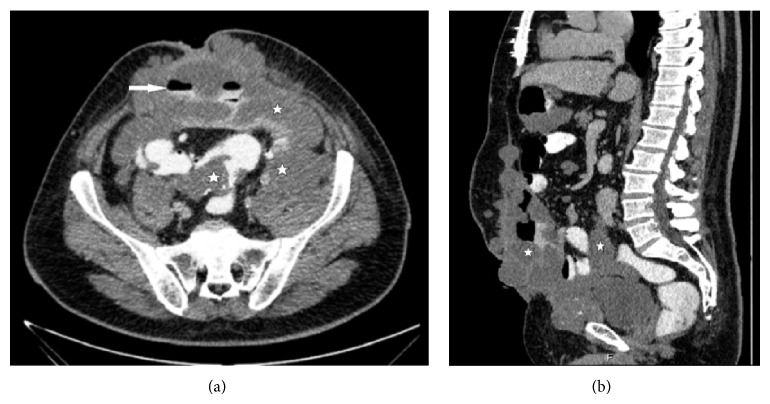
Cross-section (a) and sagittal (b) multiplanar reconstruction from CECT. These images demonstrate that the tumor creates air-fluid levels (arrow) by wrapping segments of intestine (stars) and extends from the anterior of the bladder to the anterior wall of the abdomen.

**Figure 3:**
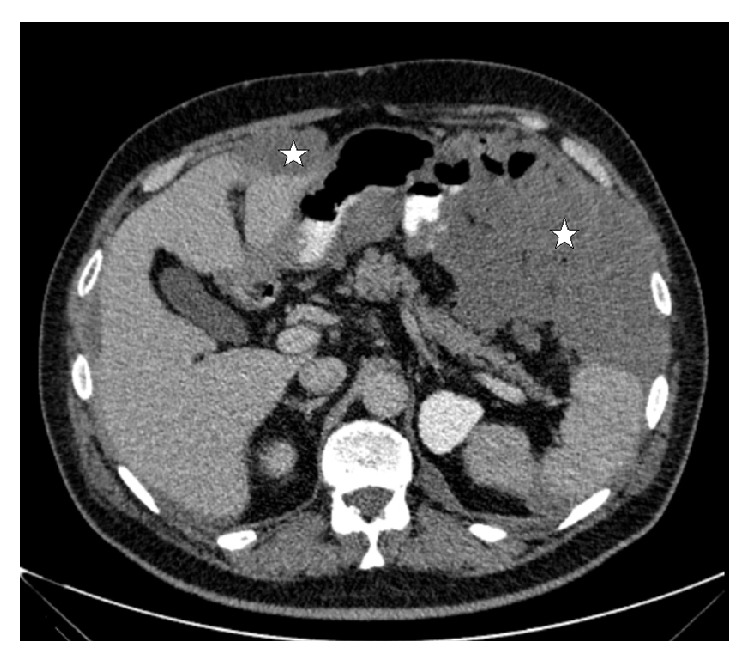
This CECT image shows metastatic implantations (stars) disseminated across serosal surfaces in the intraperitoneal area.

**Figure 4:**
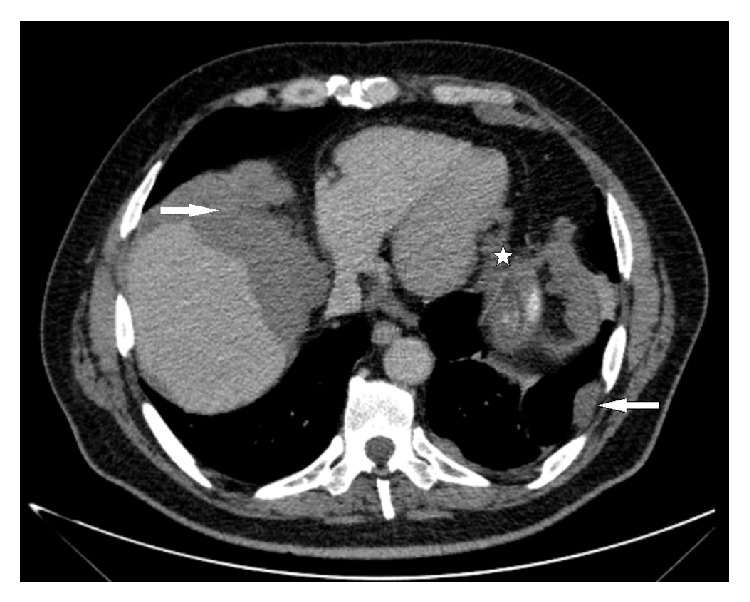
Metastatic nodules disseminated on pleural surfaces (arrows) and pericardial fat pads (star) are visualized by CECT.

A 61-year-old male was admitted to our urology department with complaints of abdominal pain, hematuria, a known mass in the abdomen, and discharge of fluid from the umbilicus. He had been evaluated 25 years ago for macroscopic hematuria, and an urachal tumor was identified. Transurethral resection of the bladder tumor (TUR-BT) revealed mucinous adenocarcinoma of the urachus, and a partial cystectomy was performed. After the operation, the patient was noncompliant with the recommended follow-up protocol. Twenty years later, he was examined by a urologist for the same complaint. Cystoscopy and TUR-BT confirmed the same diagnosis. Thoracoabdominal computed tomography (CT) verified a local recurrence of urachal carcinoma, but revealed no distant metastases. Radical cystectomy was recommended to the patient, but he did not consent. The patient underwent a second partial cystectomy at another hospital 6 months later but the umbilical remnant was not resected at that time. Three months later, local recurrence was identified, and local excision of both tumor and umbilicus was performed. Two years later, intraperitoneal invasion had occurred and the patient underwent a total colectomy. All laboratory findings were normal except for hematuria. In radiological investigation, a CT scan revealed a massive lesion filling the bladder lumen with extension to the anterior wall of the abdomen, a heterogeneous internal structure, and amorphous punctate calcifications (Figure 1). The lesion was approximately 20 cm in size and formed an air-fluid level at the umbilicus. The central aspect of the lesion had a necrotic appearance. The tumor extended to the level of the symphysis pubis. There was a mass surrounding and wrapping the rectum, the sigmoid colon, and the small intestine, causing narrowing in several areas (Figures 2(a) and 2(b)). An enterocutaneous fistula connected to the mass was seen. There were metastatic implantations, as well as ascites in the peritoneum, which had spread on the serosal surfaces (pseudomyxoma peritonei) (Figure 3). Disseminated metastases were observed in intra- and extraperitoneal areas, on pleural surfaces, and on the pericardial fat pads. Suspicious lesions concordant with potential metastases were also found in the basal segments of the left lung (Figure 4).

The patient was deemed to be inoperable and chemotherapy was subsequently initiated. The patient ultimately was treated symptomatically, as he was unable to tolerate the chemotherapy, and died 6 months later.

## 3. Discussion

Urachal diseases may be congenital or acquired [2]. While the main etiology of acquired urachal diseases is infection and urachal carcinoma, congenital diseases may develop. These depend upon the type of defect and are formed in part due to partial or total closure of the urachus. There are four known types of congenital pathologies: patent urachus, urachal cyst, urachal sinus, and vesicourachal diverticulum [2].

The development of malignancy in urachal remnants is typically seen in or after middle age [3]. Clinical prognosis of urachal malignancies is generally worse than those of primary bladder carcinomas, because they are rarely operable, with delayed diagnosis due to nonspecific symptoms, location, and a lack of clinical findings. Most cases show local invasion or metastatic disease at the time of diagnosis. The differential diagnosis of urachal tumors and primary bladder neoplasms may be difficult. Ninety percent of urachal carcinomas occur at the bladder side of the urachus, originating from the level of the dome, and the malignancy progresses through to the urachal side. Though there is a tendency for extravesical spread, primary bladder neoplasms mostly initiate from the apex and spread into the lumen [2].

Ultrasonography is the first choice for imaging in the diagnosis of urachal malignancy, because of its widespread availability, ease of use, and inherent suitability in the evaluation of cystic lesions. Uncomplicated cases may often be diagnosed with routine abdominal ultrasound alone. Such malignancies may be found on the anterosuperior surface of the bladder, with midline placement, and appear elliptic and hypoechoic in structure. However, cystography or sinography may often be required for final diagnosis [4].

CT and magnetic resonance imaging (MRI) are beneficial in monitoring tumor progression, identifying both extension and spreading, as well as for preoperative surgical planning. However, in the case of complicated cystic lesions or contrast-enhancing lesions, it may be extremely difficult to differentiate between infected urachal remnants and urachal carcinomas. Cysts with mural nodularity and calcification in contrast-enhanced computed tomography (CECT), as well as hematuria and the absence of findings of infection in the perilesional soft tissues, are usually signs of malignancy [2]. However, it should be considered that perilesional irregularity and alterations in fatty tissue planes may also be seen in infections. A tru-cut biopsy or cyst aspiration may ultimately be required for the final diagnosis [5].

Despite the fact that a normal urachus is wrapped in transitional epithelium, urachal carcinomas occur as adenocarcinomas in roughly 90% of cases [6]. Consistent with other mucinous adenocarcinomas, typical psammoma calcifications may be seen. Calcifications are present in 50–70% of cases and may be punctate or peripheral [7]. Calcifications are noted at the midline and in supravesical masses and are diagnostic to a large extent of urachal carcinomas. In CT evaluation, urachal carcinomas are found to be solid, cystic, or a combination of both [2]. In 60% of cases, there are low-intensity components in the lesion due to the presence of mucin [7].

In the case of urachal carcinoma, there is no consensus over the implementation of partial cystectomy of the dome of the bladder versus radical cystectomy; for both there is a consensus on the removal of urachus and umbilicus [5, 7]. Carcinomas may develop from urachal remnants that have not been completely cleared. Metastasis occurs first at the level of the pelvic lymph nodes. Subsequently, systemic metastases are noted in the bladder, intestines, other pelvic structures, lungs, liver, brain, and bones [8, 9].

Bruins et al. stated that the peritoneum is the location where distant metastasis is most commonly seen, starting at the age of approximately 45 years [1]. While initial metastasis in the present case was seen at the intraperitoneal level, it was discovered at a much older age. When our case was initially identified, only a partial cystectomy was performed. In spite of this, the surgery was followed without symptoms for approximately 20 years. After that time, the tumor recurred locally; as the patient would not consent to a radical cystectomy, he was treated with two more partial cystectomies and one local excision. The median survival in metastatic cases is reported to be between 12 and 24 months [10, 11]; it was approximately 3 years in this case. Median overall survival is between 37 and 48 months in the case literature [1, 12]. The 25-year-long survival of this metastatic case without any standard surgical or oncological treatment is of interest.

Urachal carcinomas are usually aggressive tumors but may also present as slow growing and locally recurrent. Therefore, correct radiological assessment of patients is important. Surgical treatment should include partial or radical cystectomy, as well as excision of the urachus and umbilicus to prevent local recurrence and distant metastasis.
